# Eight Reasons for Planting an Orchard

**Published:** 1861-07

**Authors:** 


**EIGHT REASONS FOR PLANTING AN ORCHARD.**— 1. Dr. Dwight used to remark to his pupils at Yale, that the raising of fruit was the cheapest and pleasantest way of entertaining one's friends. We are creatures of society, and it is a very important object to make the social board attractive to all who honor us with their friendship. A dish of well-grown apples is always wholesome and acceptable.

2. An orchard is an ornament to the farm, beautiful in its spring blossoms, its summer drapery of green, and its autumn burden of yellow and ruddy fruit. No farm is complete without its acres of orchard.

3. The cultivation of fruit is a very pleasant occupation, and has an important influence upon the mind and heart of the cultivator. It requires higher intelligence than the growing of the annual crops. It fosters forecast and hopefulness, and tends to a cheerful temper.

4. It makes home attractive—children are universally fond of fruit, and the home where this luxury is always enjoyed, will be more loved on that account. It will be in pleasant contrast with many homes around them.

5. It will tend to guard children against vice and crime. So strong is the desire for fruit, that they may steal it if it be not provided for them at home. And the boy that grows up plundering his neighbor's fruit-yard and orchard, is very likely to steal more valuable things when he becomes a man.

6. It is a very sure investment. An apple-tree, if well planted, is about as hardy as an oak, and sure to bear fruit according to the labor bestowed upon it. When houses burn up, and banks fail, and railroad stocks depreciate, the orchard will yield dividends.

7. It is not only a sure investment for yourself, but for your children. No real-estate in their inheritance is likely to be so permanently valuable. An orchard in good soil will bear fruit for a hundred years.

8. It is a perpetual incitement to thanksgiving to the bountiful Creator. It yields its burden of precious fruit year after year, giving large returns for the labors of the husbandman, and calling him to behold the wisdom and goodness of providence. Do not fail to plant that long-deferred orchard, and while you are about it, select good marketable fruit. The best is the cheapest.—*American Agriculturist.*

